# Utility of Biometric Measurements from Fetal Magnetic Resonance Imaging for Improved Antenatal Diagnosis of Dandy–Walker Spectrum Posterior Fossa Lesions

**DOI:** 10.3390/diagnostics15101295

**Published:** 2025-05-21

**Authors:** Rakhee M. Bowker, Kranthi K. Marathu, Marissa Pharel, Jubril O. Adepoju, Farzan Vahedifard, Seth Adler, Mehmet Kocak, Xuchu Liu, Sharon E. Byrd

**Affiliations:** 1 Department of Pediatrics, Division of Neonatology, Rush University Children’s Hospital, Rush University Medical Center, Chicago, IL 60612, USA; 2 Department of Diagnostic Radiology and Nuclear Medicine, Rush Medical College, Rush University Medical Center, Chicago, IL 60612, USA; kranthi_k_marathu@rush.edu (K.K.M.); marissa_pharel@rush.edu (M.P.); jubril_o_adepoju@rush.edu (J.O.A.); fvahedifard@mgh.harvard.edu (F.V.); seth_adler@rush.edu (S.A.); mehmet_kocak@rush.edu (M.K.); achillesy@msn.com (X.L.); sharon_byrd@rush.edu (S.E.B.)

**Keywords:** fetal MRI, central nervous system anomalies, posterior fossa malformations, vermian hypoplasia, Blake’s pouch cyst, Dandy–Walker malformation, biometric measurements

## Abstract

**Background/Objective:** The accurate diagnosis of congenital central nervous system abnormalities is critical to pre- and postnatal prognostication and management. When an abnormality is found in the posterior fossa of the fetal brain, parental counseling is challenging because of the wide spectrum of clinical and neurodevelopmental outcomes in patients with Dandy–Walker (DW) spectrum posterior malformations. The objective of this study was to evaluate the utility of biometric measurements obtained from fetal magnetic resonance imaging (MRI) to facilitate the prenatal differentiation of Dandy–Walker (DW) spectrum malformations, including vermian hypoplasia (VH), Blake’s pouch cyst (BPC), and classic Dandy–Walker malformation (DWM). **Methods:** This retrospective single-center study evaluated 34 maternal–infant dyads referred for fetal MRI evaluation of suspected DW spectrum malformations identified on antenatal ultrasound. Radiologists took posterior fossa measurements, including the vermis anteroposterior (AP) diameter, vermis height (VH), and tegmento–vermian angle (TVA). The posterior fossa, fourth ventricle, and cisterna magna were classified as normal, large, or dilated. The postnatal imaging findings were evaluated for concordance. The acquired values were compared between the groups and with normative data. The genetic testing results are reported when available. **Results:** A total of 27 DW spectrum fetal MRI cases were identified, including 7 classic DWMs, 14 VHs, and 6 BPCs. The TVA was significantly higher in the DWM group compared with the VH and BPC groups (*p* < 0.001). All three groups had reduced AP vermis measurements for gestational age compared with normal fetal brains, as well as differences in the means across the groups (*p* = 0.002). **Conclusions:** Biometric measurements derived from fetal MRI can effectively facilitate the prenatal differentiation of VH, BPC, and classic DWM when assessing DW spectrum posterior fossa lesions. Standardizing biometric measurements may increase the diagnostic utility of fetal MRI and facilitate improved antenatal counseling and clinical decision-making.

## 1. Introduction

The accurate diagnosis of congenital central nervous system (CNS) abnormalities is critical to pre- and postnatal prognostication and management. Prenatal diagnosis of severe CNS anomalies may inform obstetric decision-making about fetal monitoring and route of delivery [1]. Additionally, fetal magnetic resonance imaging (MRI) confirmation of CNS anomalies detected on fetal ultrasound (US) prior to delivery may guide access to critical antenatal counseling, coordination of complex family-centered care for the remainder of pregnancy and delivery, and minimize separation of the mother–infant dyad, which is particularly important in the setting of life-limiting diagnoses [2]. Diagnosing CNS anomalies prior to delivery also facilitates the coordination of complex care for the infant after delivery, including delivery at tertiary care centers with access to appropriate pediatric subspecialty services. For fetal anomalies, providing information to pregnant mothers about diagnostic and therapeutic procedures that will occur in the neonatal intensive care unit (NICU) in a multidisciplinary setting prior to delivery can help relieve parental stress and anxiety [3]. When an abnormality is found in the posterior fossa of the fetal brain, parental counseling is challenging because of the wide spectrum of clinical and neurodevelopmental outcomes in patients with Dandy–Walker spectrum posterior malformations [4].

Although CNS malformations remain quite rare, the spectrum of posterior fossa malformations, including Dandy–Walker malformation (DWM), mega cisterna magna (MCM), Blake’s pouch cyst (BPC), and vermian hypoplasia (VH), affect approximately 1 in 5000 live births [5]. The term “Dandy-Walker malformation” was coined by German Psychiatrist Clemens Ernst Benda in 1954. However, the condition itself was first described by John Bland-Sutton in 1887, with further descriptions provided by Walter Dandy and Blackfan in 1914 and Taggart and Walker in 1942 [6]. The Dandy–Walker malformation (DWM) or syndrome is a posterior fossa anomaly characterized by agenesis or hypoplasia of the vermis, cystic enlargement of the fourth ventricle with communication to a large cystic dilated posterior fossa, upward displacement of the tentorium and torcula (torcular–lambdoid inversion), and enlargement of the posterior fossa [6]. Classic DWM, VH, and MCM likely represent a continuum of congenital structural abnormalities affecting the cerebellum and posterior fossa [7]. The term “Dandy-Walker variant” was originally introduced to describe milder phenotypes, but more recently has been discouraged as a diagnostic classification due to its frequent misuse; however, this non-specific term continues to appear in regular clinical practice and the literature [8,9,10].

Although DWM can occur sporadically, genetic and chromosomal abnormalities are important factors in the etiology of DWM, with abnormalities in chromosomes 3, 9, 13, and 18 most frequently linked to posterior fossa malformations [11]. Juan et al. (2024) suggested that copy number variant (CNV) testing should be the primary diagnostic method for genetic testing in isolated posterior fossa malformations, whereas a sequential approach consisting of karyotyping, CNV testing, and whole-exome sequencing (WES) should be utilized in non-isolated posterior fossa malformations [12]. Posterior fossa malformations highlight the interplay between genetic and environmental factors in abnormalities in brain development. Although most cases are sporadic, some may result from chromosomal aneuploidy; Mendelian disorders; and environmental exposures, including viral infections and exposures to teratogens [13]. For example, cerebellar hypoplasia and VH may be seen in congenital rubella syndrome, in which maternal infection with the rubella virus early in pregnancy can disrupt the normal development of the cerebellum during early fetal development. As the cerebellum is the center for motor coordination and higher cognitive functions, posterior fossa malformations may be associated with impaired spatial navigation and musical learning ability, as well as impacting motor, sensory, cognitive, emotional, and autonomic functioning [14]. Consequently, the neurodevelopmental prognosis for children with congenital posterior fossa malformations is quite broad and significantly affected by the presence of associated genetic diagnoses or syndromes. Awareness of the developmental basis of posterior fossa malformations may facilitate improved detection of morphologic changes identified on imaging and enable more accurate differentiation and diagnosis of congenital posterior fossa anomalies [13].

Recently identified radiologic features that aid in distinguishing DWM from other posterior fossa abnormalities, such as the tail sign [15], choroid plexus/tela choroidea location [16,17], and fastigial recess shape [18], have not been incorporated consistently into standard diagnostic criteria. Classic DWM, VH, and BPC result from abnormal development of the posterior membranous area and are collectively referred to as the Dandy–Walker spectrum. The rhombic lip, a dorsal stem cell zone that drives the growth and maintenance of the posterior vermis, is specifically disrupted in DWM, with reduced proliferation and self-renewal of the progenitor pool and altered vasculature [18]. In 2021, Haldipur and colleagues proposed a unified model for the developmental pathogenesis of DWM, in which a disruption in rhombic lip development during embryonic development (through either aberrant vascularization or a direct insult) causes reduced proliferation and failed expansion of the rhombic lip progenitor pool, resulting in inferior vermis hypoplasia and dysplasia [18]. In this model, the timing of the insult to the developing rhombic lip dictates the extent of hypoplasia, distinguishing DWM caused by insult prior to 14 weeks gestation (GA) from VH caused by an insult after 14 weeks GA.

When congenital CNS abnormalities are detected during antenatal ultrasonography, fetal MRI studies are utilized for diagnostic confirmation to improve prenatal counseling and planning for the appropriate postnatal management of affected infants who may require referral to a tertiary care facility for the availability of pediatric neurosurgery, pediatric neurology, and other relevant subspecialists. Recent advances in fetal MRI characterization have enhanced the delineation of posterior fossa anomalies, including the improved identification of subtle cerebellar and vermian anomalies and their clinical implications [19]. Evaluating the structures in the posterior fossa through fetal MRI and categorizing the malformations into clinically relevant groups using routine measurements may provide high utility for accurately diagnosing these relatively rare CNS anomalies and providing better prognostication for the family and the multidisciplinary team caring for the infant after birth [20,21]. At this time, the literature on biometric measurements using fetal MRI in pregnancies affected by suspected congenital Dandy–Walker spectrum anomalies is quite limited.

The primary objective of this study was to evaluate the utility of biometric measurements obtained from fetal MRI to facilitate the prenatal differentiation of Dandy–Walker spectrum lesions. By examining and quantifying specific anatomical features, the authors aimed to determine whether these measurements could aid in distinguishing between VH, BPC, and classic DWM during routine clinical evaluations. The improved ability to reliably differentiate between these diagnoses antenatally may enable more accurate prognostication by maternal fetal medicine and fetal neonatal medicine providers and facilitate the delivery of infants with DWM at tertiary care facilities with appropriate resources to improve the care of these vulnerable patients and their families, while minimizing unnecessary separation of mothers and infants in the NICU for those infants with more benign findings.

## 2. Materials and Methods

### 2.1. Study Population and Design

This was a retrospective study that involved a retrospective single-center review of a convenience sample of cases of posterior fossa malformations confirmed by fetal MRI at the Rush University Medical Center (RUMC) between 2004 and 2020. The sole inclusion criterion was a referral to the RUMC for fetal MRI based on concern after a fetal ultrasound for a CNS anomaly with fetal MRI confirmation of posterior fossa malformation. By using relevant keywords, a chart review was performed and a comprehensive list of all fetal MRIs conducted at the study center was generated, which resulted in the identification of 200 fetal MRIs through the key search. Preliminary CNS anomalies noted on routine prenatal ultrasounds were reviewed by MFM physicians. The MRI studies were retrospectively examined by the research radiologists, who excluded MR studies without a posterior fossa abnormality. Mothers referred with concern for posterior fossa malformations on fetal USs who were not able to undergo fetal MRI prior to delivery due to claustrophobia or maternal contraindications to undergoing MR scans were also excluded. The diagnostic quality of the imaging was determined by the neuroradiologists. Concordance between the fetal US, fetal MRI, and postnatal imaging was also evaluated when available. Clinical information was collected through a retrospective chart review that utilized the Rush Fetal and Neonatal Medicine Center QI database and the electronic medical record (EMR).

This study was approved by the Rush University Institutional Review Board, and informed consent was waived given the retrospective nature of this study with all this study’s data de-identified and all fetal MRI scans obtained for routine clinical purposes based on usual and customary care.

To ensure unbiased evaluations, two independent board-certified pediatric neuroradiologists (S.E.B. and M.K. with over 15 years clinical experience), who were not aware of the participants’ clinical evaluations or the initial interpretations of the scans, reviewed the fetal MRI images. The neuroradiologists were able to establish concordance in 92% of cases. In cases of any disagreements between the two neuroradiologists, a final diagnosis was achieved by their consensus. The interobserver agreement statistics showed kappa statistics between 0.90 and 1 for the presence of DWM, VH, and BPC. This rigorous process helped ensure the objectivity and reliability of this study’s findings.

### 2.2. Data Collection and Management

Chart reviews were performed, and the following data were extracted from the EMR: maternal age at delivery, maternal comorbid diagnoses, maternal parity, maternal race/ethnicity, mean GA in weeks at the fetal MRI, route of delivery, known teratogenic exposures during pregnancy, amniocentesis results if performed, birth GA (completed gestational weeks), sex, and postnatal length of stay (LOS) in the General Care Nursery (GCN) or NICU after delivery. The pregnancy outcomes were categorized as termination of pregnancy, spontaneous intrauterine fetal demise, inborn live birth, or death in the neonatal period. In this study, clinical information was obtained from the pediatric neurology and genetics clinic when available. Genetic evaluation included when available, including karyotyping, chromosomal microarray analyses, and whole-exome sequencing (WES).

### 2.3. Scanning Parameters for Fetal Magnetic Resonance Imaging

At the study institution, fetal MR scans were conducted without maternal sedation using an Espree 1.5 T MR scanner (Siemens Medical Solutions, Erlangen, Germany) using a single-shot turbo spin-echo (HASTE) T2-weighted pulse sequence. The parameters included time to echo (TE): 120 milliseconds (ms); repetition time (TR): 4300 ms; field of view (FOV): 23 (phase) × 23 (frequency) cm; slice thickness (ST): 3 mm; interslice spacing (IS): zero; and matrix: 256 (phase) × 180 (frequency) in the axial, sagittal, and coronal projections. HASTE refers to Half Fourier Single-shot Turbo spin-Echo, a single-section T2-weighted MR sequence that acquires images in less than one second.

### 2.4. Classification of Posterior Fossa Malformations on Fetal MRI

Two independent board-certified neuroradiologists (S.E.B. and M.K. with over 15 years of experience) reviewed all the fetal MR brain images of the participants while blinded to the clinical history. These qualified experts reviewed all the images using a Picture Archiving and Communication System (PACS) workstation, which is a specialized tool for managing and interpreting medical images. Each patient was categorized into one of three groups by the radiologists: BPC, VH, or classical DWM.

The morphological classification of posterior fossa malformations was originally proposed by Barkovich et al. in 1989 [22] and was subsequently refined by Klein et al. in 2003 [23], who introduced specific radiological criteria for diagnosing DWM [24]. To be classified as having classical DWM in this study, fetuses needed to meet three specific imaging criteria: (1) cystic enlargement of the fourth ventricle, (2) partial or total absence of the vermis, and (3) an enlarged posterior fossa with upward displacement of the tentorium and torcula.

The cases were categorized as VH when the smaller-than-normal vermis demonstrated a high tegmento–vermian angle (TVA) greater than 18 degrees, without any expansion of the posterior fossa. To receive a BPC diagnosis, the vermis demonstrated a size typical for gestational age, with a TVA greater than 18 degrees [21]. In this study, the radiologists measured several aspects of the posterior fossa using PACS craniocaudal (CC) height and anterior-to-posterior (AP) measuring devices. Measurements included the vermis, AP pons, and size of the largest lateral ventricle. These measurements were recorded in millimeters (mm) and rounded to the nearest mm following the methodologies described by Garel et al. in 2005 [25] and Tilea et al. in 2009 [26] for the assessment of certain parameters and the techniques outlined by Chapman et al. in 2018 [27] for evaluating the TVA and superior posterior fossa angle. The measurements obtained from the fetal MRI were compared with the normal values reported by Kline-Fath and colleagues (2020) [28].

By employing these standardized measurement techniques, the radiologists aimed to gather precise data to aid in the differentiation and characterization of the various posterior fossa anomalies within the Dandy–Walker continuum. Regarding the cisterna magna, the radiologists encountered difficulties in obtaining repeatable measurements due to vermian rotation and distortion. As a result, a cisterna magna was categorized as either normal or enlarged based on the radiologists’ visual assessment.

To validate the prenatal diagnosis of each patient, the researchers examined the postnatal brain MRI results whenever available to evaluate concordance.

Throughout this study, all authors remained blinded to the patient identifiers, and strict confidentiality was maintained regarding protected health information. The final diagnosis was arrived at by consensus.

### 2.5. Statistical Analysis

Data were obtained and entered into Microsoft Excel LTSC MSO (Version 2401 Build 16.0.17231.20194) 64-bit. De-identified data were analyzed utilizing SPSS^®^ Statistics software version 26 (IBM, Armonk, NY, USA). The concordance between the fetal US, fetal MRI, and postnatal imaging was assessed. Student *t*-tests were used for comparisons of the two groups, and ANOVAs were utilized for the comparisons of three or more groups. A *p*-value of 0.05 or less was considered statistically significant. Parametric tests were conducted for normally distributed data. Means and their corresponding standard deviations or median interquartile ranges are reported for quantitative variables. Frequencies are presented as percentages to represent qualitative variables.

## 3. Results

After a review of 200 fetal MRIs from pregnant women evaluated by the Fetal and Neonatal Medicine Center for suspected fetal CNS anomalies, a total of 34 maternal–infant dyads were identified that met this study’s inclusion criteria for fetal MRI to evaluate for concerns of posterior fossa abnormalities diagnosed on antenatal ultrasounds. Of the 34, 27 maternal–infant dyads were identified with fetal MRI findings consistent with DWM, VH, or BPC.

The remaining maternal–infant dyads were found to have non-Dandy–Walker spectrum conditions, including an isolated enlarged or prominent cisterna magna or other CNS anomalies, as listed in Table 1.

The mean gestational age (GA) at the time of the fetal MR imaging was 26 gestational weeks. Among the Dandy–Walker spectrum cases, 7 cases were diagnosed with classic DWM, 13 cases were categorized as VH, and 6 cases were classified as BPC. This study’s findings revealed that the cisterna magna was enlarged in 100% (7/7) of patients with DWM, which was a higher frequency compared with the VH group (50%, 7/14) and the BP group (50%, 3/6), as indicated in Table 2. The concordance of the prenatal US, fetal MRI, and postnatal imaging are reported in Table 3.

**Figure 1:**
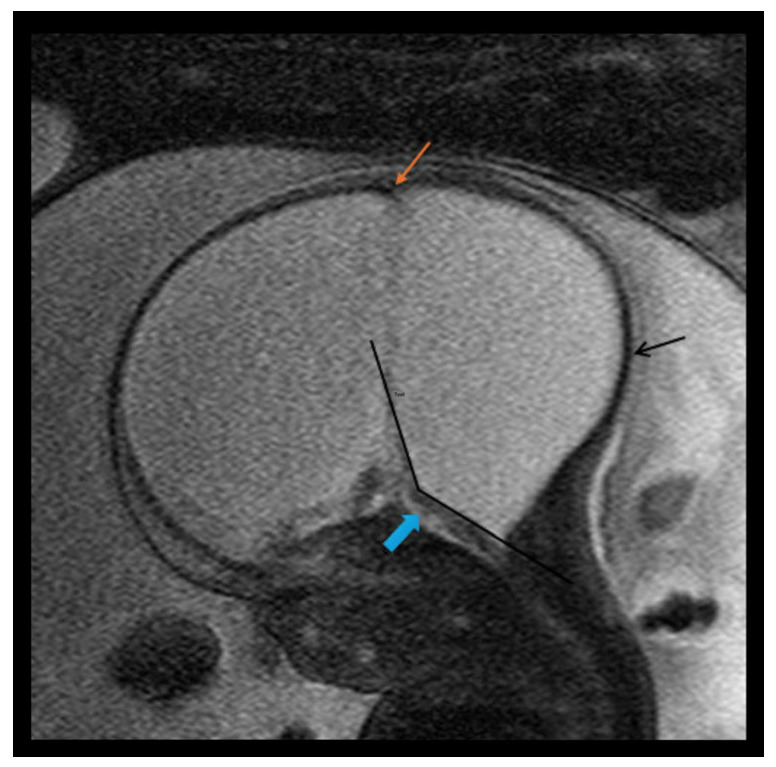
Fetal MRI image of a fetus at 31 gestational weeks with a classical Dandy–Walker cyst and massive hydrocephalus with a possible form of encephaloclastic schizencephaly. The T2-weighted sequence in the sagittal plane depicts a large posterior fossa cyst representing massive enlargement of the 4th ventricle as part of a large classical Dandy–Walker cyst. Pictured is an enlarged intracranial cavity occupied almost completely by CSF, a large posterior fossa cyst with ballooning of the occiput (black arrow), and a thin brainstem (blue arrow) with anterior displacement. There was no evidence of a cerebellum and there was elevation of torcular implantation (orange arrow) and an increased tegmento–vermian angle (black lines). There were enlarged lateral ventricles secondary to the massive hydrocephalus and/or a form of encephaloclastic schizencephaly.

**Figure 2:**
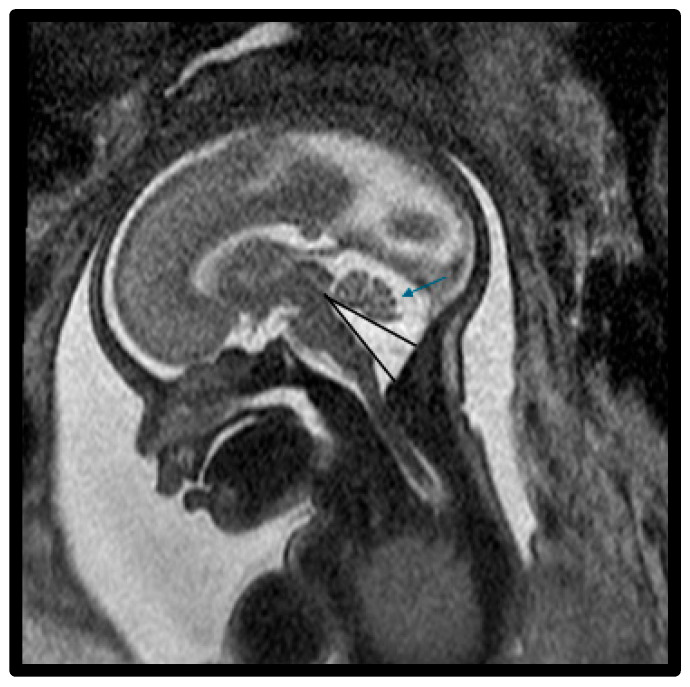
Fetal MRI image of a fetus at 25 gestational weeks with vermian hypoplasia. The midline T2-weighted sagittal image shows an enlarged fourth ventricle, decreased vermian biometry, and enlarged fluid space of the posterior fossa. There was mild hypoplasia of the postero-inferior vermis with slight upward rotation (blue arrow) and increased tegmento–vermian angle (black lines). The fastigial recess was shallow and there was mild widening of the vallecula, the midline outlet of the fourth ventricle. There was a prominent and abnormal fourth ventricle with a loss of the normal diamond shape. The fourth ventricle is shown inferiorly connecting with a prominent cisterna magna through the vallecula.

**Figure 3:**
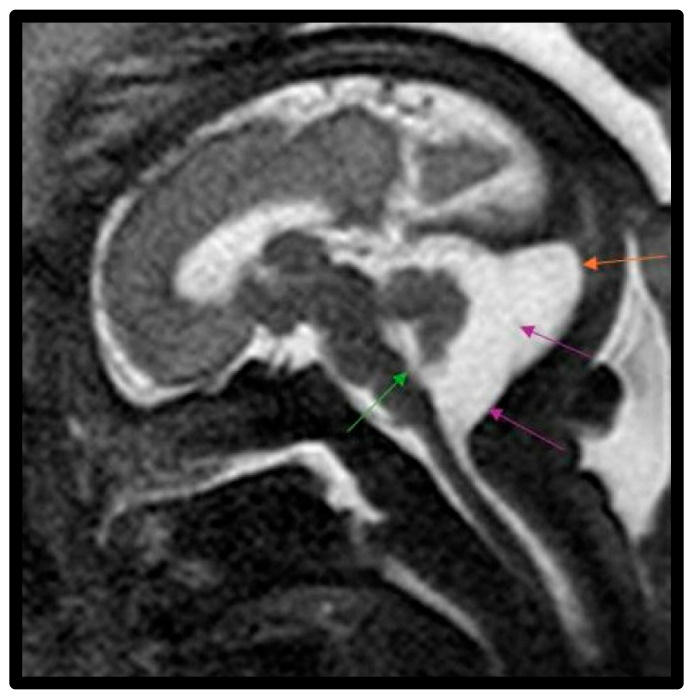
Fetal MRI image of a fetus at 33 gestational weeks with a Blake’s pouch cyst. The T2-weighted sequence in the sagittal plane depicts a slight widening of the midline outlet of the 4th ventricle (green arrow). There is a membrane within the midportion of the CSF space of the cisterna magna with slight bowing of this membrane posteriorly, which is consistent with a Blake’s pouch cyst (purple arrows) involving posterior fossa within this enlarged cisterna magna (orange arrow). The cyst freely communicated with the fourth ventricle with upward displacement of the vermis and elevation of the tentorium.

**Figure 4:**
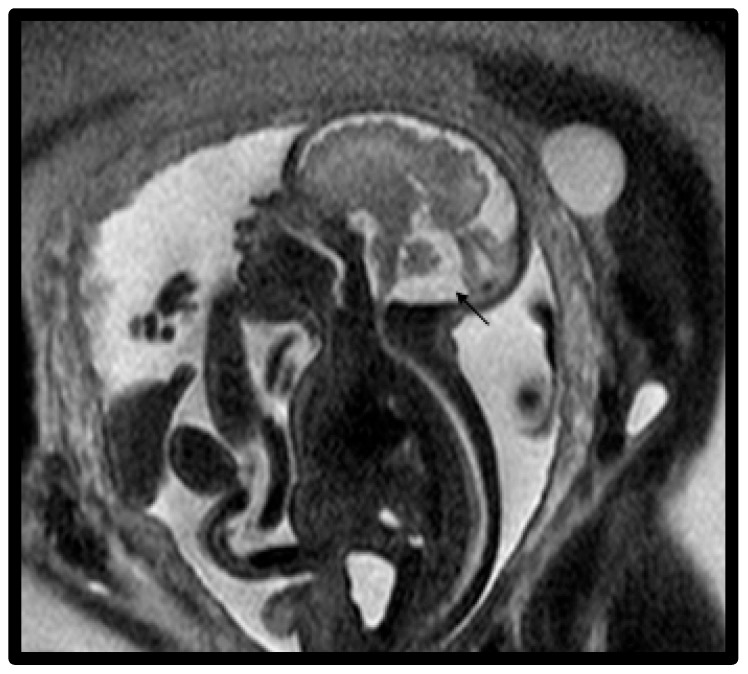
Fetal MRI image of a fetus at 32 gestational weeks with an enlarged cisterna magna. The midline T2-weighted sagittal image shows an enlarged cisterna magna (1.2–1.4 cm) and increased CSF subarachnoid spaces overlying the cerebellum (black arrow). The fourth ventricle did not communicate with the cisterna magna and there was a normally inserted torcula.

In this study, a total of 27 maternal–infant dyads were found to have Dandy–Walker spectrum posterior fossa malformations on the fetal MRI. Six had abnormal results from genetic testing, with an additional infant diagnosed with congenital CMV and another with maternal Coxsackie infection (Table 4). Abnormal genetic testing results in the case series are detailed as follows, with additional details of the postnatal course in Table 4. Figure 1 depicts a fetal MRI of a preterm infant with classic DW cyst. Figure 2 depicts a fetal MRI image of a fetus at 25 gestational weeks with vermian hypoplasia. Figure 3 depicts a fetal MRI image of a fetus at 33 gestational weeks with a Blake’s pouch cyst. Figure 4 depicts a fetal MRI image of a fetus at 32 gestational weeks with an enlarged cisterna magna.

❖ Case 1 with DWM was noted to have hydrocephalus, markedly elevated CPK, bilateral talipes equinovarus deformity, overlapping fingers, a vertebral anomaly consisting of fusion of the second and third ribs, and retinal dysgenesis and was clinically diagnosed with Walker–Warburg syndrome.
❖ Case 7 with DWM had a postnatal microarray, which identified a pathogenic 4.1 Mb loss at 3q24q25.1, including Z1C1 and Z1C4, which has been proposed as a critical region for brain anomalies. The entire deleted interval included 32 genes, of which 8 (Z1C1, RNF13, MED12L, HPS3, GYG1, CP, CRLN1, P2RY12) are associated with known clinical disorders.
❖ Case 8 with DWM had one pathogenic change in ASPMc.7783_7786del, p.Lys2595Tyrfs 20 (AR primary microcephaly); a hetA VUS change in TUBGCP4 (AR) c.602G>A, p.Gluy201Asp; and a het A VUS change in MAPK8IP (AD) c.1057G>C, p.Asp353His, het identified.
❖ Case 14 with VH was found to have a duplication of material on the long arm of chromosome X, including part of the FMR1 and AS1-FMR1 genes. In general, a loss of function of the FMR1 gene leads to Fragile X syndrome. It is not clear whether disruption of the gene would lead to a partial or complete loss of FMR1 function, especially since the disruption resulted in a duplication. In this patient, the CNS findings, including microcephaly, were explained by congenital CMV, as urine CMV testing on admission was positive with >3 million copies.
❖ Case 19 with VH was diagnosed with Trisomy 13.
❖ Case 31 with VH, cerebellar hypoplasia, moderate dilation of the lateral ventricles, and mild-to-moderate dilation of the 3rd ventricle was diagnosed with Smith–Lemli–Opitz Syndrome. Additional abnormalities for this infant included balanced AV canal Rastelli type 1 with no coarctation, posterior cleft palate, optic nerve dysplasia, infantile cataracts of both eyes, hearing loss, left talipes equinovarus, bilateral hand and foot polydactyly, a right hip dislocation, and restrictive lung disease due to scoliosis.

## 4. Discussion

In our study, we evaluated maternal–infant dyads whose pregnancy was complicated by a fetus with a Dandy–Walker spectrum CNS anomaly diagnosed on fetal MRI, including 7 with classic DWM cyst, 14 with VH, and 6 with BPC. The objective of this study was to assess the effectiveness of biometric measurements obtained from fetal MRI in the evaluation of posterior fossa lesions, specifically those along the Dandy–Walker spectrum. We conducted a retrospective single-center review of 200 mothers referred for fetal MRI evaluation due to suspected CNS anomalies, with 34 referred with concern or posterior fossa malformations. Of these, we included 27 with Dandy–Walker spectrum anomalies with fetal MRIs of diagnostic quality. In our cohort, we found that the TVA was significantly higher in the DWM group than in the VH and BP groups (Table 2, *p* < 0.001), as well as when compared with commonly used fetal imaging metrics [21,22,23,24,25,26,27,28]. The anterior-to-posterior (AP) vermis measurements were significantly different between the groups (Table 2, *p* < 0.002), and we observed reduced anterior-to-posterior (AP) vermis measurements in all three groups when compared with normal fetal brains [29]. Regarding cisterna magna (CM) enlargement, all patients with DWM and half of the patients with VH and BPC subtypes exhibited CM enlargement. The percentage of patients with CM enlargement across the three groups was significantly different (Table 2, *p* < 0.002), suggesting a potential association between CM enlargement and the severity of the Dandy–Walker spectrum.

Fetal MRI is an interactive scanning of the moving fetus that is becoming an increasingly valuable noninvasive tool for evaluating fetal abnormalities [30,31]. Due to the low prevalence of fetal CNS abnormalities, many community obstetricians performing screening ultrasounds have limited exposure to congenital brain abnormalities [32]. Thus, for suspected fetal CNS anomalies identified on routine antenatal ultrasound, fetal MRI has become an essential method for evaluating the fetal brain, facilitating a diagnostic confirmation and improved prognostic information regarding fetal CNS anomalies and permitting improved parent counseling and determination of the delivery location at tertiary care centers for affected patients with access to appropriate pediatric subspecialists, including pediatric neurosurgery and neurology services [33]. The MERIDIAN diagnostic accuracy study evaluated 570 fetuses to diagnose fetal developmental brain abnormalities utilizing fetal MRI [34]. The investigators found that US and fetal MRI have absolute diagnostic accuracies of 68 and 93 percent, respectively. With increasing gestational age, the disparity between US and fetal MRI increased. Pregnant mothers tolerated the procedure well, with 95% of the responders saying they would have fetal MRI again in a similar situation. According to health professional interviews, fetal MRI was acceptable to physicians and considered beneficial as a supplement to US, but not as a replacement. When compared with US alone, fetal MRI resulted in a higher cost. Additionally, there may be reporting bias from referring clinicians on diagnostic and prognostic outcomes. The authors suggested that using fetal MRI as an adjuvant to US enhances the diagnostic accuracy and confidence in the diagnosis of prenatal brain disorders [34]. In our cohort, there was not a significant difference between the accuracy of the fetal US and fetal MRI for the diagnosis of posterior fossa lesions (Table 3).

Accurately detecting posterior fossa anomalies along the Dandy–Walker spectrum before birth presents distinct clinical implications for the postnatal management of these infants, as well as the delivery location and urgency of postnatal imaging. DW spectrum disorders can have a wide spectrum of clinical and neurodevelopmental outcomes requiring pediatric subspecialty care, including neonatology, neurology, pediatric radiology, neurosurgery, genetics, physical therapy, and speech language pathology. When community obstetricians identify concern for a CNS abnormality on routine screening, referral to an MFM for confirmatory testing is warranted. Fetal MRI confirmation of posterior fossa abnormalities identified on prenatal ultrasound can facilitate the antenatal counseling of parents and permit the coordination of care to facilitate delivery at tertiary or quaternary care centers with access to pediatric subspecialists. In our cohort, classic DWM was associated with unifying genetic diagnoses or syndromes in three out of nine patients (33%), including a diagnosis of Walker–Warburg syndrome (Case 1 in Table 3 and Table 4), which is notable for microcephaly, overlapping fingers, bilateral talipes equinovarus deformities, hypotonicity with absent suck/swallow, and retinal dysgenesis. This infant was discharged with home hospice on TPN via a Broviac catheter due to oral feeding intolerance. A second infant (Case 7 in Table 3 and Table 4) with classic DWM was noted to have a pathogenic 4.1 Mb loss at 3q24q25.1, including ZIC1 and ZIC4 genes, a critical region for brain anomalies that explains the infant’s clinical findings. A third infant with classic DWM (Case 8 in Table 3 and Table 4) was found to have a pathogenic change in the ASPM gene, which was inherited in an autosomal recessive fashion and has been associated with primary microcephaly, along with several VUSs. A normal karyotype was identified in four of the nine infants with classic DWM and no genetic testing was available for two outborn infants. Four out of nine of our cohort with classic DWM required a VP shunt placement in infancy or early childhood.

For infants with imaging consistent with VH in our cohort, genetic testing or screening was normal in 8 out of 17 cases. No testing was available for five infants. One patient with VH (Case 14, Table 3) had congenital CMV with associated severe microcephaly, a blueberry muffin rash, calcifications on the head imaging, retinitis, and lower extremity spasticity. This patient also had a duplication of material on the long arm of chromosome X, including part of the FMR1 and AS1-FMR1 genes, although congenital CMV infection was likely the etiologic environmental exposure for this infant. One patient with VH (Case 19, Table 3 and Table 4) was found to have Trisomy 13, which is known to be associated with posterior fossa malformations. One interesting case (Case 23, Table 3 and Table 4) of VH was associated with a heterozygous VUS in the BRAF gene, as well as titers concerning for maternal Coxsackie virus infection during pregnancy. This infant had an associated right ectopic kidney in the iliac fossa; two VSDs; a supernumerary nipple; and multiple midline lesions, including epulis of the gum, a cyst on the lip, and right-sided sensorineural hearing loss.

Clinically, MCM and arachnoid cysts are usually considered incidental findings, whereas cysts in the DW spectrum are associated with other developmental anomalies of the cerebellar hemispheres and vermis and often present with hydrocephalus early in life [35]. A mega CM communicates freely with both the fourth ventricle and subarachnoid space. The arachnoid mater, the middle layer of the meninges, is a thin, avascular membrane that separates the dura mater and pia mater, with the subarachnoid space containing CSF in between. A posterior fossa arachnoid cyst represents a CSF collection within duplicated layers of the arachnoid, which may communicate directly with the subarachnoid space. Both the MCM and arachnoid cysts may exert mass effects on the cerebellum or progress to hydrocephalus, but less frequently than in DW spectrum disorders.

Within the radiological spectrum of posterior fossa cysts and cyst-like malformations, BPC is a distinct entity with clinical signs and symptoms that may range from asymptomatic to hydrocephalus [35,36,37]. Isolated BPC is radiologically characterized by a normal-sized vermis with an elevated TVA. A fastigial angle measurement on fetal MRI may be a reliable adjunct in distinguishing BPC from VH, especially when the vermis size is borderline [38]. Children with isolated BPC generally have a favorable neurodevelopmental prognosis; however, close monitoring by the pediatrician and pediatric neurosurgery is advised due to the possibility of postnatal obstructive hydrocephalus requiring shunting [36,37]. One case series of six children with BPC emphasized their initial radiological uniformity coupled with widely variable clinical presentations. This case series included one patient who died in infancy after developing marked hydrocephalus with high intracranial pressure, leading to massive cerebral ischemia in the setting of hemorrhagic BPC, which was complicated by vitamin K deficiency from biliary atresia [36]. A previously healthy child with isolated BPC presented at 2 years of age with gait impairment from progressive hydrocephalus that was corrected with shunting via an endoscopic third ventriculostomy [36]. This case series also included a previously healthy patient who presented at age 69 years when he developed gait disturbance, memory impairment, dysphasia, headaches, nausea, blurred vision, and opisthotonus. This patient was ultimately diagnosed with BPC complicated by obstructive hydrocephalus, which required treatment with an endoscopic third ventriculostomy. Other patients with BPC have compensated hydrocephalus that may not require neurosurgical treatment, although continued close monitoring is advised. Accordingly, infants diagnosed using fetal imaging with BPC will benefit from proactive monitoring by their pediatrician and neurosurgery, allowing for timely neurosurgical intervention when needed and watchful waiting for those infants and children whose head circumferences stabilize with the normal progression of developmental milestones. In our cohort, the infants diagnosed with BPC all had normal genetic testing.

The long-term neurodevelopmental consequences of isolated VH remains poorly defined, with inconsistencies in the classification of cerebellar anomalies further obscuring the prognostic picture. In one cohort, infants with isolated posterior fossa abnormalities diagnosed on fetal MRI typically had normal neurological outcomes, while those with additional anomalies demonstrated higher morbidity and mortality [14]. In another recent cohort study, fetuses with classic DWM were more likely to have associated CNS anomalies and required more postnatal intervention(s) compared with those diagnosed with BPC or VH [11]. A study that evaluated neurodevelopmental outcomes in patients diagnosed with isolated VH on fetal MRI indicated that although children with postnatally confirmed isolated inferior VH had an overall lower mean developmental performance compared with infants with normal postnatal MRI, all children were free of major neurodevelopmental impairment and disability, suggesting a relatively benign outcome at preschool age [39]. A follow-up study evaluated these children at school age and found that 17/20 children with postnatal confirmation of isolated VH had normal cognitive, language, social, and behavioral outcomes [40]. The authors found that more extensive cerebellar malformations or chromosomal anomalies were strongly associated with worse neurodevelopmental outcomes in these patients [40]. Despite the benign functional outcome of children with isolated VH, parents of these children carry an elevated and enduring burden of stress.

In our cohort, as in the literature [23,41,42], the infants with more extensive cerebellar malformations and associated genetic diagnoses had a substantially higher burden of morbidity and mortality. In this series of 34 mother–infant dyads referred with concern for posterior fossa abnormalities on fetal imaging, we identified six such cases with DW spectrum malformations associated with genetic diagnoses, including Walker–Warburg syndrome, Smith–Lemli–Opitz syndrome, and Trisomy 13. Congenital CMV infection was suspected as the underlying etiology in one patient in our cohort. Pathogenic deletions and duplications in critical regions for brain anomalies were also identified. As the availability of rapid whole-genome genetic testing improves, correlating the results of genetic testing with the DW spectrum phenotype on fetal MRI may enable more accurate prognostication. A sequential genetic testing strategy—starting with chromosomal microarray and copy number variant (CNV) testing followed by whole-exome sequencing (WES) in non-isolated posterior fossa malformations—significantly improves the diagnostic yield and is recommended for optimal prenatal genetic diagnosis. In a study by Zhang et al., this approach led to a 47.5% detection rate in non-isolated PFMs, while isolated cases had a lower yield, supporting a tiered testing strategy [12]. Postnatal brain MRI for the confirmation of antenatal findings remains critical for infants diagnosed with DW spectrum anomalies on fetal imaging, as false positive abnormal fetal MRI results have been reported in the literature, particularly with isolated VH [8].

The present study significantly contributes to the existing body of literature by reporting on the biometric measurements of the posterior fossa obtained from fetal MR imaging in a substantial sample of patients with confirmed diagnoses of malformations involving posterior fossa along the Dandy–Walker spectrum. Moreover, the inclusion of postnatal imaging validation and clinical follow-up enhances the significance and clinical relevance of our findings. Improving the accuracy of fetal diagnostics may enable better patient counseling and management approaches, as well as the appropriate utilization of resources for patients who require close multidisciplinary follow-up given the risk of progressive hydrocephalus and neurodevelopmental impairment. Emerging artificial intelligence tools are being developed to automate the segmentation of fetal brain structures on MRI, which may improve the diagnostic reproducibility in posterior fossa malformations and enable the early prediction of postnatal neurodevelopmental outcomes [43]. Additionally, the need for genetic testing to identify associated genetic syndromes can permit more accurate prognostication for these patients and their families and alleviate some of the uncertainty that contributes to increased stress and anxiety for caregivers of these children.

This study contributes to the limited literature on fetal MR imaging biometric assessments in patients with posterior fossa abnormalities. The standardization of these measurements can facilitate the routine clinical practice of evaluating posterior fossa features on prenatal MR imaging. The sample size in this study was small, and therefore, additional research with larger sample sizes is essential to confirm and validate these findings.

### Study Limitations

We wish to acknowledge several limitations that could impact the interpretation and generalizability of these findings. The myriad terminologies used in the context of the Dandy–Walker spectrum raise concerns about the applicability of our findings to clinical practice beyond our institution. To reduce confusion and enhance precise prognostication, certain fetal imagers have suggested refraining from using the term “Dandy-Walker variant” and instead considering a small and rotated vermis as an imaging phenotype rather than a primary diagnosis. This approach is particularly relevant when considering genetic abnormalities, as genetic mutations have been found to be more commonly associated with cerebellar hypoplasia than with a classic Dandy–Walker malformation (DWM). Furthermore, there is a suggestion that the severity of imaging findings in the posterior fossa alone may not always accurately correlate with the severity of the clinical phenotype [8].

One major limitation was the retrospective design of this study, which introduced inherent biases and restricted the internal validity. Moreover, this study’s single-institution nature and the temporal constraints of data collection may limit the external validity of the findings. Considering the acknowledged variability and overlapping imaging findings within the Dandy–Walker spectrum, the diagnostic categories utilized in this study might be subject to scrutiny, particularly due to inconsistent terminology and criteria used in the existing literature.

## 5. Conclusions

In conclusion, this study emphasized the potential value of biometric measurements derived from fetal MRI when assessing and confirming posterior fossa lesions along the Dandy–Walker spectrum detected on prenatal ultrasound. We found that biometric measurements derived from fetal MRI could effectively facilitate the prenatal differentiation of VH, BPC, and classic DWM when assessing DW spectrum posterior fossa lesions. Standardizing biometric measurements may increase the diagnostic utility of fetal MRI in clinical practice and facilitate improved antenatal counseling and clinical decision-making when fetal CNS anomalies are suspected. Further research with larger sample sizes and longitudinal neurodevelopmental outcome data is warranted to validate these findings and advance our understanding of fetal brain development and the neurodevelopmental implications of posterior fossa lesions.

## Figures and Tables

**Table 1:** Birth data, sociodemographic characteristics, and clinical course.

Fetuses with Suspected Posterior Fossa Lesions Referred for Fetal MRI	
**Maternal and Birth Characteristics**	
Maternal age at delivery (years), mean ± SD	27 ± 6
Gestational age (weeks) at fetal MRI, mean ± SD	26.5 ± 5.8
Maternal comorbid diagnoses, *N* (%)	
Preeclampsia	1 (3%)
Gestational or chronic hypertension	5 (15%)
Gestational diabetes	3 (9%)
Teen pregnancy	3 (9%)
Advanced maternal age	4 (12%)
Race/ethnicity, *N* (%)	
White	9 (26.5%)
Black	16 (47%)
Hispanic	9 (26.5%)
Other	0 (0%)
Cesarean delivery, *N* (%)	9 (26.5%)
Apgar score	
1 min, median (25th, 75th)	8 (4, 9)
5 min, median (25th, 75th)	9 (9, 9)
Birth order	
Singleton	34 (100%)
Multiple gestation	0 (0%)
Insurance	
Commercial	16 (47%)
Medicaid	17 (50%)
Unknown or no insurance	1 (3%)
Teratogenic exposures during pregnancy	4 (11.8%)
Amniocentesis	
Normal karyotype and microarray	13 (38%)
Abnormal results	1 (3%)
Birth weight (g), mean ± SD	2892 ± 558
Birth gestational age (weeks), mean ± SD	37 ± 4
Male sex, *N* (%)	16 (47%)
**Clinical Characteristics and Hospital Course**	
Fetal MRI findings ^b^	
Isolated prominent or enlarged cisterna magna	5 (15%)
Blake’s pouch cyst	6 (18%)
Classic Dandy–Walker cyst	7 (21%)
Vermian hypoplasia	13 (38%)
Arachnoid cyst	1 (3%)
Encephalomalacia or schizencephaly	1 (3%)
Dysgenesis of the corpus callosum with colpocephaly	1 (3%)
Imaging characteristics	
Postnatal imaging confirmed fetal MRI findings ^a^	20 (87%)
Postnatal imaging findings disagreed with fetal MRI findings	3 (13%)
VP shunt placement	
During NICU admission	3 (9%)
After NICU discharge	1 (3%)
Seizure disorder	2 (6%)
Mode of feeding at NICU discharge (*N* = 18)	
PO ad lib on demand	14 (78%)
Gastrotomy tube feedings	3 (17%)
Parenteral nutrition	1 (6%)
Congenital infection	
CMV	1 (3%)
Coxsackie	1 (3%)
Postnatal genetic testing	
Normal	13 (38%)
Abnormal ^c^	6 (18%)
Not sent/unknown	15 (44%)
Disposition	
Inborn live birth	22 (65%)
Outborn	12 (35%)
Interruption of pregnancy based on fetal MRI findings	1 (3%)
IUFD	2 (6%)
NICU LOS (day), median (25th, 75th)	8 (3, 32)
Live birth with death before NICU discharge, *N* (%), *n* = 22	4 (18%)

Abbreviations: CMV: cytomegalovirus, g: grams, IUFD: intrauterine fetal demise; LOS: length of stay; MRI: magnetic resonance imaging, NICU: neonatal intensive care unit, PO: per os. ^a^ Postnatal imaging was not available in 12 cases due to IUFD (2), interruption of pregnancy (1), or delivery outside hospital (9). ^b^ Percentiles will add up to more than 100% because frequency of abnormalities is reported. ^c^ Abnormal genetic testing or genetic diagnosis was identified in 6 patients in this series.

**Table 2:** Posterior fossa measurements for Dandy–Walker spectrum disorders.

	DWM (*n* = 6) ^#^	VH (*n* = 14)	BPC (*n* = 6)	*p*-Value
Vermis height (mm)	5.0 ± 2.6 (*n* = 6)	6.9 ± 2.8	9.5 ± 2.7	0.09 (NS)
Vermis AP (mm) *	10.8 ± 6.2 (*n* = 6)	11.8 ± 3.0	14.8 (SD, 3.3)	0.002
TVA (°) *	93.7° ± 18.1° (*n* = 6)	50.9° ± 17.3°	21.7° (SD, 15.5°)	<0.001
Enlarged CM *	7/7 (100%)	7/14 (50%)	3/6 (50%)	0.002

Data are presented as the mean ± SD or *n* (%). * A *p*-value of < 0.05 was considered statistically significant. ^#^ For one case diagnosed with DWM, the vermis could not be visualized, which resulted in the inability to assess the vermis height, AP vermis, and TVA for this case. Consequently, 6 DWM patients are presented in Table 2 instead of 7. Abbreviations: AP: anterior to posterior, DWM: classic Dandy–Walker malformation, VH: vermian hypoplasia, BPC: Blake’s pouch cyst, TVA: tegmento–vermian angle, CM: cisterna magna, mm: millimeters.

**Table 3:** Concordance of prenatal US, fetal MRI, and postnatal imaging findings.

Case	Prenatal US Findings	Fetal MRI Findings	Postnatal Imaging
1	Suspected DWM	Classic DWM	Classic DWM
2	Suspected DWM	Classic DWM	Not available
3	Hydrocephalus, large DWM, large supratentorial cyst, very little brain tissue remaining, and no evidence of cerebellar tissue.	Classic DWM	Large DW cyst occupying posterior half of the intracranial cavity. In the supratentorial region, there was a large cystic structure that represented a combination of the lateral ventricles and third ventricle under pressure from massive hydrocephalus. Small rim of cortical cerebral tissue along interior and lateral aspect of the supratentorial region. Thin brainstem.
4	Hydrocephalus, CNS anomaly.	DWM, massive DW cyst, encephalomalacia vs. schizencephaly.	Not available
5	CNS anomaly	Classic DWM, colopocephaly, dysgenesis of the CC, nodular heterotopia, holoprosencephaly.	CNS malformations include ACC and cerebellar abnormalities.
6	DWM with retrocerebellar DW cyst, mild supratentorial hydrocephalus.	Classic DWM	DWM and thin CC
7	Macrocephaly and DWM	Classic DWM	Classic DWM
8	DWM, absent CC, clubfeet, and SUA.	Classic DWM with ACC	Classic DWM, ACC with colpocephaly and migrational abnormalities involving the bifrontal parietal regions, and diffusely gracile brainstem.
9	Hydrocephalus	DWM with no thalamus or spinal cord.	Not available due to IUFD
10	CNS anomaly	VH	VH
11	BPC vs. VH vs. DWM	VH	Not available
12	Suspected VH vs. DWM	VH	VH
13	Suspected DWM	VH	Not available due to IUFD
14	Congenital CMV and suspected DWM.	VH	VH and calcifications and diffuse marked brain parenchymal loss from congenital CMV.
15	FGR, enlarged CM, VH	VH	Not available
16	Suspected DWM with partial absence of inferior portion of the posterior lobe of the vermis with mild-to-moderate widening of the vallecula.	VH	VH
17	Suspected DWM	VH	Mild prominence of 4th ventricle and CM. Normal ventricles. Repeat HUS was normal.
18	Dilated posterior fossa, absent cerebellar vermis, micrognathia, absent nasal bone.	VH	VH, hypoplasia of inferior cerebellar vermis, mild prominence/enlargement of 4th ventricle, mild prominence/enlargement of the vallecula, absence of the inferior aspect of the vermis of the cerebellum, and a small retrocerebellar cyst vs. enlargement of the CM. Normal posterior fossa structures including normal cerebellum and brainstem. Normal spine.
19	Suspected common atrium, perimembraneous VSD (AVSD), and DW spectrum malformation.	VH	DCC and DW variant, mineralizing vasculopathy of the basal ganglia.
20	Absence of inferior vermis. Fourth ventricle slightly enlarged and opened into a retrocerebellar cyst vs. CM consistent with DW variant. Cerebellar hemispheres were small but appeared normal for gestational age. Normal supratentorial region, fetal spine, and spinal cord.	VH	Not available
21	DW variant with cerebellar hypoplasia vs. isolated cerebellar hypoplasia.	VH	Not available
22	Suspected CNS anomaly	VH	VH, brain MRI showed partial aplasia of the cerebellum and vermis consistent with a very mild DW variant.
23	Mild inferior VH, right renal agenesis vs. right ectopic kidney, absent nasal bone, small pericardial effusion and LV hypertrophy, possible VSD.	VH	VH with DWM, hypoplastic cerebellum
24	Enlarged CM	BPC	Not available
25	BPC vs. enlarged CM	BPC	BPC
26	BPC vs. enlarged CM	BPC	Enlarged CM
27	Suspected bilateral polymicrogyria with enlarged CM and BPC, ventricles on the upper limit of normal, brain appears dysmature for stated age, and progressive IUGR.	BPC with polymicrogyria	Not available
28	Bilateral CDH with liver up, IUGR, BPC with no associated mass effect of hydrocephalus, no cerebellar VH, and high likelihood of ECMO due to severe pulmonary hypoplasia and risk of pulmonary hypertension.	BPC	Postnatal imaging was not obtained due to the severity of CDH.
29	Arachnoid cyst	Arachnoid cyst	Large PF cyst with mild mass effect on the cerebellar hemispheres, normal-appearing vermis and vallecula, and elevation of torcula consistent with arachnoid cyst within the CM. Subcutaneous fluid collection in the occipital region, which measured 7 mm in maximal width. Bilateral closed lip schizencephaly with polymicrogyria. Increased cortical gray matter with small sulci along portions of the bilateral frontal lobes, consistent with polymicrogyria. No hydrocephalus.
30	Enlarged CM	Enlarged CM	Not available
31	Suspected DWM, left talipes equinovarus, and AVSD.	Enlarged CM	Postnatal imaging was not consistent with DWM, mild dilation of lateral ventricles and superior 3rd ventricle, and cerebellar hypoplasia with prominent inferior basal cisterns.
32	Enlarged CM	Enlarged CM	Not available
33	Enlarged CM	Enlarged CM	Normal brain MRI
34	Hydrocephalus, polycystic kidney, two-vessel umbilical cord.	Enlarged CM	Colpocephaly, absence of the septum pellucidum, mild fullness of the 3rd ventricle, posterior fossa fluid collection, and left occipital echogenicity. Brain MRI showed marked dilatation of the lateral ventricle, mild dilatation of the 3rd ventricle with no septum pellucidum and vein CC, mega CM, possible hypoplastic cerebellum, left-sided parietal occipital infarct.

Abbreviations: ACC: agenesis of the corpus callosum, AVSD: atrioventricular septal defect, CC: corpus callosum, CM: cisterna magna, CMV: cytomegalovirus, DWM: Dandy–Walker malformation, DCC: dysgenesis of the corpus callosum, ECMO: extracorporeal membrane oxygenation, HUS: head US, LV: left ventricle, MRI: magnetic resonance imaging, SUA: single umbilical artery, VSD: ventricular septal defect.

**Table 4:** Case series with genetic testing and clinical course.

Case	PFM Type on fMRI	Additional Imaging Findings	GA at fMRI	Genetic or Unifying Diagnosis	Pregnancy Outcome	Brief Narrative Summary of Newborn Clinical Course
1	Classic DWM	Moderate to severe hydrocephalus, thin CC, hypoplastic cerebellum, large PF.	34	Walker-Warburg syndrome	Live birth, discharged with home hospice.	Term male, C/S delivery, admitted to NICU for 34 days. Microcephalic, overlapping fingers, bilateral talipes equinovarus deformity, hypotonicity, absent suck/swallow, decreased reflexes, dysmorphic features, retinal dysgenesis, reduced life expectancy. Discharged with home hospice and TPN via Broviac due to feeding intolerance. VP shunt placed.
2	Classic DWM	No hydrocephalus, normal cerebellum, mild DW cyst.	25	----	Outborn	Postnatal course not available due to outborn status.
3	Classic DWM	Absent cerebellum, large PF, dilated 4th ventricle, enlarged CM, severe hydrocephalus, massive DW cyst, small and thin brainstem.	31	Normal karyotype and microarray	Live birth, died before discharge.	Preterm C/S delivery, intubated in DR due to poor tone, no respiratory effort, central cyanosis, bradycardia, and massive macrocephaly. EEG showed global neuronal dysfunction. Imaging showed hydranencephaly. Respiratory failure with apnea and respiratory acidosis. Infant was compassionately extubated and died on day of life 7. Autopsy showed macrocephaly, posterior fossa cyst, severe cerebellar hypoplasia and partial hypoplasia of the brainstem, severe hydrocephalus with severe atrophy of cerebellar cortices, and no deep gray matter structures identified. Abnormal heart congenital polyvalvular disease moderate type was diagnosed with stenosis of tricuspid, mitral and aortic valves, and atypical hypertrophic cardiomyopathy. Liver with islands of extramedullary erythropoiesis.
4	Classic DWM	Absent cerebellum, enlarged PF, dilated 4th ventricle, enlarged CM, severe hydrocephalus, massive DW cyst, encephalomalacia or schizencephaly, hypoplastic brainstem, thin cerebral hemispheres.	33	----	Outborn	Postnatal course not available due to outborn status.
5	Classic DWM	Normal cerebellum, enlarged PF, normal 4th ventricle, enlarged CM, normal torcula, mild-to-moderate hydrocephalus, DCC, colpocephaly, DW cyst, nodular heterotopia, holoprosencephaly.	19	Normal karyotype and microarray	Interruption of pregnancy with D&E at 20 weeks gestation.	Autopsy showed SUA, CNS malformations, as described prenatally, including ACC and cerebellar abnormalities, and postaxial polydactyly of the right hand and right foot.
6	Classic DWM	Hypoplastic vermis & cerebellum, enlarged PF, dilated 4th ventricle, enlarged CM, normal torcula, mild supratentorial hydrocephalus, DWM with retrocerebellar DW cyst, aqueductal stenosis, thinning of CC, absent CSP.	21	Normal karyotype and microarray	Live birth	Admitted to NICU with hydrocephalus in the setting of congenital DWM. No seizures during NICU admission. VP shunt placed for hydrocephalus. Discharged home on PO ad lib feedings.
7	Classic DWM	Severely hypoplastic cerebellum, enlarged PF, dilated 4th ventricle, enlarged CM, elevated torcula, moderate hydrocephalus.	31	Pathogenic 4.1 Mb loss at 3q24q25.1, including ZIC1 and ZIC4, a critical region for brain anomalies that explained infant’s clinical findings. Deleted interval included 32 genes, 8 of which (ZIC1, RNF13, MED12L, HPS3, GYG1, CP, CRLN1, P2RY12) are associated with known clinical disorders.	Live birth	C/S delivery, admitted to NICU. Postnatal imaging confirmed large posterior fossa DWM. VP shunt placed for hydrocephalus. Discharged home on PO ad lib feedings with anti-reflux formula. No seizures during NICU admission.
8	Classic DWM	Hypoplastic cerebellum, enlarged posterior fossa, enlarged 4th ventricle, enlarged CM, normal torcula, no hydrocephalus, DWM cyst, ACC.	25	Seizure panel identified 3 VUS changes in FASN, SLC12AS, and SLC6A. Brain malformation panel identified pathogenic change in ASPM c.7783_7786del, p.Lys2595Tyrfs 20 (AR primary microcephaly); het A VUS change in TUBGCP4 (AR) c.255C>A, p.Tyr85; het A VUS change in B9D1 (AR) c.602G>A, p.Gluy201Asp; het A VUS change in MAPK8IP (AD) c.1057G>C, p.Asp353His, het.	Live birth	C/S delivery. Infant with clubfeet and single umbilical artery. LOS was 36 days in the NICU. Seizures, inability to feed safely by mouth requiring GT placement, cardiomyopathy requiring heart failure medications. Postnatal imaging consistent with areas of heterotopia superimposed on DWM with ACC and mild left hydronephrosis. VP shunt placed for hydrocephalus.
9	Classic DWM	Hydrocephalus.	16	Normal karyotype	IUFD	Postnatal radiograph confirmed stillborn female with bell-shaped thorax and multiple congenital anomalies.
10	VH	Prominent CM. No cerebellar hypoplasia. Remote microhemorrhages in left cerebellar hemisphere.	19	No testing available	Live birth	NSVD with unremarkable newborn nursery course, discharged home with normal neurological exam at nursery discharge.
11	VH	Flattened globes.	22	No testing available	Live birth	Live birth at OSH per maternal records, postnatal course not available in EMR.
12	VH	No hydrocephalus.	30	Normal karyotype and microarray	Live birth	Prolonged NICU hospitalization with VH confirmed. Severe disabilities, including right facial deformity with malformed ear and facial droop, respiratory failure requiring tracheostomy and mechanical ventilation, gastrostomy tube dependence, hearing impairment, and vision impairment.
13	VH	---------------------------	22	Normal karyotype and microarray	IUFD	IUFD in the setting of FGR with teratogenic maternal exposure to methimazole during pregnancy and maternal Graves’ disease.
14	VH	Calcifications in the basal ganglia, thalami, and diffuse grey matter, including parenchymal and lateral ventricular wall calcifications consistent with congenital CMV. Marked ventriculomegaly suggestive of brain volume loss. Abnormal cortical development and migration. Intraventricular hemorrhage with fluid levels within bilateral lateral ventricles and 4th ventricle and cystic lesions.	30	Congenital CMV. Genetic testing noted duplication of material on the long arm of chromosome X, including part of the FMR1 and AS1-FMR1 genes.	Live birth	Congenital CMV. Coagulopathic at birth with a diffuse rash, calcifications on head US, retinitis, and microcephaly (<1st %ile). Neurological exam notable for lower extremity spasticity. Passed newborn hearing screen.
15	VH	Hypoplastic vermis, enlarged CM without cerebral ventriculomegaly. Fetal imaging notable for echogenic bowel, globular and thickened placenta suggestive of fetal infection vs. chromosomal anomaly, fetal echo with normal cardiac structure.	21	No testing available	Outborn	Outborn infant, postnatal course not available in EMR.
16	VH	Partial absence of inferior portion of vermis with mild-to-moderate widening of vallecula. Normal 4th ventricle. Postnatal brain MRI confirmed mild inferior VH, mild superior rotation of vermis with a normal-sized PF.	36	Normal prenatal screening	Live birth	C/S delivery with benign postnatal course. Normal neurological exam. Discharged home with mother on day of life 3 from the general care nursery feeding PO ad lib.
17	VH	Postnatal HUS showed prominence of 4th ventricle and CM with normal lateral ventricles, cerebellar vermis not well visualized.	20	Normal prenatal screening, CMV and toxoplasmosis testing were negative.	Live birth	C/S delivery, admitted to the NICU. CONS bacteremia. Normal eye exam without chorioretinitis. Passed hearing screen. Infant had normal growth and development at routine well-child checks through 15 months of age.
18	VH	Fetal US with dilated PF, absent cerebellar vermis, absent nasal bone, micrognathia. Postnatal HUS showed hypoplasia of inferior cerebellar vermis, enlarged 4th ventricle. Postnatal brain MRI showed mild enlargement of 4th ventricle, absent inferior aspect of cerebellar vermis, and small retro-cerebellar cyst with enlargement of CM. Normal brainstem and cerebellum.	25	Normal karyotype and microarray	Live birth	C/S delivery, unremarkable newborn nursery course with normal neurological exam. Well-child exams through to age 11 years with normal neurological exam with normal strength, tone, and developmental milestones.
19	VH	Postnatal HUS showed mineralizing vasculopathy in basal ganglia (can be seen in aneuploidy). Possible dysgenesis of the CC and hypoplastic vermis. Rectangular fluid-filled space between the brainstem and cerebellum continuous with expected location of 4th ventricle. Incomplete anterior portion of the cerebellum at the midline.	23	Trisomy 13	Live birth	C/S delivery. Severe PPHN that required high-frequency oscillatory ventilation. Significantly constricted distal pulmonary arteries with no surgical repair possible. Congenital heart deformity with common atrium, large VSD, bicuspid aortic valve, large PDA. Polydactyly of left foot with supernumerary digit at lateral foot attached at 5th toe near MTP joint that contained 2 hypoplastic phalanges. Chromosomes confirmed Trisomy 13. Infant died before NICU discharge after compassionate redirection of care.
20	VH		19	No testing available	Live birth	Liveborn female based on maternal records but infant’s postnatal records not available in EMR.
21	VH		21	No testing available	Outborn	Postnatal course not available
22	VH	Postnatal head US showed no IVH or ventricular dilation with possible deficiency of the cerebellar vermis. Brain MRI showed partial aplasia of cerebellum and vermis.	22	Normal male karyotype (46 XY) on amniocentesis, FISH was normal.	Live birth	NSVD due to maternal preterm labor at 31 weeks, 41-day NICU stay with benign course, room air, PO ad lib feedings, normal developmental exam, passed hearing screen.
23	VH	Prenatal imaging showed mild inferior VH, right renal agenesis vs. right ectopic kidney, absent nasal bone, small pericardial effusion and LV hypertrophy, possible VSD. Postnatal imaging showed VH and hypoplastic cerebellum.	29	Amniocentesis normal karyotype & microarray. Noonan panel with heterozygous variant of uncertain significance in BRAF gene identified, c.786A>G, pGln262=. Amniotic fluid with higher titer for Coxsackie virus (1:8) consistent with resolving infection.	Live birth	Term male C/S delivery at term, admitted to NICU for 18-day LOS, discharged in room air, PO ad lib feeding. Postnatal imaging confirmed VH, right ectopic kidney in the iliac fossa, two tiny VSDs, ventral hernia, multiple midline lesions including epulis of gum, cyst on lip, bifurcated gluteal folds, asymmetry of testes, supernumerary nipple, closed left clavicular fracture. Right-sided sensorineural hearing loss.
24	BPC	Fetal US concerning for enlarged CM, found to be BPC on fMRI.	36	No testing available	Outborn	Postnatal course not available
25	BPC	Fetal US with enlarged CM vs. BPC, found to be BPC on fMRI, postnatal HUS showed BPC.	34	cfDNA low risk for aneuploidy or sex chromosome disorders.	Live birth	C/S delivery, 4-day LOS, phototherapy for hyperbilirubinemia, discharged home with mother from GCN feeding PO ad lib in room air. Postnatal HUS showed BPC.
26	BPC	Fetal US with MCM vs. BPC. FMRI suggestive of BPC. Postnatal brain MRI most consistent with MCM.	23	cfDNA low risk for aneuploidy or sex chromosome disorders.	Live birth	C/S delivery, unremarkable newborn course, discharged home from GCN on DOL 2 feeding PO ad lib in room air. Postnatal imaging consistent with MCM.
27	BPC	Fetal US with FGR, suspected bilateral polymicrogyria with enlarged CM and BPC, ventricles upper limit of normal, brain dysmature for stated age.	31	Declined amniocentesis, low risk quad screen.	Live birth	Apgar scores 8 and 9, admitted to the NICU, details of hospital course unavailable in EMR.
28	BPC	Fetal US notable for FGR, BPC, bilateral CDH with pulmonary hypoplasia, estimated lung volumes ~20% of expected.	25	Amniocentesis with normal karyotype and microarray.	Live birth	C/S delivery, postnatal course complicated by severe CDH with pulmonary hypoplasia, death in NICU within 24 h of birth after redirection to comfort care.
29	CM	Enlarged CM on fetal US confirmed on fetal MRI.	30	No testing available.	Outborn	Postnatal course not available.
30	MCM + CNS anomalies	Prenatal US with MCM, arachnoid cyst, absent CSP, hydrocephalus, SUA, polycystic kidney. Postnatal HUS: absent CSP, colpocephaly, mild 3rd ventricle dilation, PF fluid collection, left occipital echogenicity. Postnatal brain MRI showed marked lateral ventricle dilation, mild 3rd ventricle dilatation, absent CSP, MCM, possible hypoplastic cerebellum, left parietal–occipital infarct.	32	Amniocentesis with normal karyotype and microarray.	Live birth	NSVD, LOS was 6 days in NICU, diagnosed with left MCDK, absent CSP, DCC, hypoplastic cerebellum, left partial occipital cerebrovascular accident, MCM, bilateral optic nerve hypoplasia, left T4 hemivertebrae with mild levoscoliosis. Treated with phototherapy for hyperbilirubinemia. VP shunt placed by NSGY at 2 months of age as an outpatient. VP shunt placed for hydrocephalus.
31	MCM	Prenatal US with left talipes equinovarus, AVSD, possible DW spectrum disorder. Postnatal head CT showed moderate dilatation lateral ventricles, moderate 3rd ventricle dilation, mild 4th ventricle enlargement. No obstructive hydrocephalus. Prominent cortical sulci of both cerebral hemispheres. Prominent CSF space but no mass effect. Cerebellar hypoplasia with prominent inferior basal cisterns. Cortical volume loss and atrophy in the setting of Smith–Lemli–Opitz syndrome.	36	Amniocentesis with 46XX, diagnosed with Smith–Lemli–Opitz syndrome in infant of a mother with gestational DM requiring insulin.	Live birth	NVSD with NICU LOS was 102 days. Smith–Lemli–Optiz syndrome. No seizures. Balanced AVSD Rastelli type 1, cleft palate, left talipes equinovarus, bilateral hand and foot polydactyly, and hyponatremia. Medical NEC. CPAP for poor aeration and respiratory acidosis. GT placed for feeding challenges. Intubated for heart failure, treated with Lasix, Digoxin, and Captopril with improvement. Hypertension due to autonomic instability. Global developmental delay (non-verbal and non-ambulatory). Failed hearing screen. Scoliosis, right hip dislocation, and wheelchair bound. Infantile cataracts of both eyes, post-bilateral cataract extraction with intraocular lens implantation. Slow photoreceptor recovery time, optic nerve demyelination, and optic nerve hypoplasia. Restrictive lung disease due to scoliosis. Death at age 9 years from sepsis.
32	Enlarged CM	Enlarged CM on fetal US, confirmed on fetal MRI.	26	No testing available	Outborn	Postnatal course not available
33	Enlarged CM	Postnatal brain MRI showed normal brain with marginal CM enlargement.	32	No testing available	Live birth	Normal postnatal brain imaging, normal neurological exam at birth, discharged home from GCN after uneventful newborn nursery course.
34	Arachnoid cyst	Postnatal brain MRI notable for arachnoid cyst and bilateral closed lip schizencephaly with polymicrogyria.	33	Low risk cfDNA, microarray showed: arr(122)x2, (XY)x1. Extended contiguous regions of allele homozygosity (ROH > 8 Mb) in multiple chromosomes consistent with common descent, 50.35 Mb total (1.89% of autosomal genome). No known consanguinity but from same village in Mexico. ROH Bp linear position: chr1:159775332-169090660. chr1:229984949-238623222. chr4:165411055-181814242. chr20:41192992-57181430. Congenital brain malformation panel with multiple VUS as follows: LAMB1 (AR) c.4156CA, p.Gln1386Lys, heterozygous VUS. RAB3GAP2 (AR) c.1609GA, p.Val537Ile, Heterozygous VUS. TMEM231 (AR) c.757GA, p.Gly253Arg, heterozygous VUS. PCLO (AR) c.13434GT, p.Glu4478Asp, heterozygous VUS.TMEM67 (AR) c.167510dup, intronic, heterozygous VUS.	Live birth	NVSD, briefly admitted to the NICU with transient tachypnea of the newborn requiring CPAP but was quickly weaned to room air and transferred to the GCN. Discharged home on DOL 3 in room air, feeding PO ad lib.

Abbreviations: ACC: agenesis of the corpus callosum, AP: anteroposterior, BPC: Blake’s pouch cyst, CC: corpus callosum, CM: cisterna magna, CDH: congenital diaphragmatic hernia, CMV: cytomegalovirus, C/S: Cesarean section, DCC: dysgenesis of the corpus callosum, DW: Dandy–Walker, DWM: Dandy–Walker malformation, DM: diabetes mellitus, FGR: fetal growth restriction, fMRI: fetal magnetic resonance imaging, g: grams, GCN: general care nursery, HUS: head ultrasound, IUFD: intrauterine fetal demise, LOS: length of stay, LV: left ventricle, Mb: megabase, mm: millimeters, MCDK: multi-cystic dysplastic kidney, MCM: Mega Cisterna Magna, MRI: magnetic resonance imaging, NICU: neonatal intensive care unit, NSVD: normal spontaneous vaginal delivery, OSH: outside hospital, PDA: patent ductus arteriosus, PF: posterior fossa, PO: per os, SD: standard deviation, SUA: single umbilical artery, TVA: tegmento-vermian angle, US: ultrasound, VH: vermian hypoplasia, VPS: ventriculoperitoneal shunt, VSD: ventricular septal defect, VUS: variant of unknown significance.

## Data Availability

The data presented in this study are available on request from the corresponding author (with data availability limited by patient privacy).
